# Oxidative Stress in Atopic Dermatitis

**DOI:** 10.1155/2016/2721469

**Published:** 2016-02-23

**Authors:** Hongxiu Ji, Xiao-Kang Li

**Affiliations:** ^1^Incyte Diagnostics, 1280 116th Avenue NE, Bellevue, WA 98004, USA; ^2^Division of Transplantation Immunology, National Research Institute for Child Health and Development, Tokyo, Japan

## Abstract

Atopic dermatitis (AD) is a chronic pruritic skin disorder affecting many people especially young children. It is a disease caused by the combination of genetic predisposition, immune dysregulation, and skin barrier defect. In recent years, emerging evidence suggests oxidative stress may play an important role in many skin diseases and skin aging, possibly including AD. In this review, we give an update on scientific progress linking oxidative stress to AD and discuss future treatment strategies for better disease control and improved quality of life for AD patients.

## 1. Introduction

Atopic dermatitis (AD) or atopic eczema is a chronic relapsing inflammatory skin disease. Its prevalence is continuously increasing, affecting up to 25% of children and 2-3% of adults [1]. It is clinically manifested by itching and scratching, dry skin, patchy eczema especially on flexural locations, exudation, and skin thickening and discoloration. AD has an early onset, usually in infancy or early childhood. It may regress spontaneously after puberty in some patients, but wax and wane for life in many others. The current standard treatment for AD includes moisturizing lotions and creams, topical corticosteroids, and calcineurin inhibitors [2]. For severe cases or in acute exacerbation phase, systematic agents are often efficacious, including oral corticosteroids, cyclosporine, methotrexate, mycophenolate, and azathioprine [1]. Several biologic drugs have become available in recent years, mainly monoclonal antibodies against interleukin 4-receptor, immunoglobulin (Ig) E, and activated T or B cells [3, 4].

The pathogenesis of AD is complex and still poorly understood. In addition to genetic predisposition attributed to immune dysregulation and hypersensitivity, development and maintenance of AD are thought to be associated with environmental and psychological triggers and skin barrier defects [1, 2]. Genetic predisposition is obvious in AD patients, who often have a personal or familial history of other allergic diseases, such as asthma and allergic rhinitis. Mutation of several genes has been implicated in the systemic “atopic” immune response, characterized by a Th2 dominance and elevated IgE levels, such as IL-4, IL-4 receptor, and IL-13, or altered cutaneous inflammation, such as mast cell chymase [5]. In addition, the mutations in the filaggrin gene and the SPINK5 (serine protease inhibitor kazal-type 5) gene are associated with defective epidermal differentiation and skin barrier formation [5].

**Figure 1:**
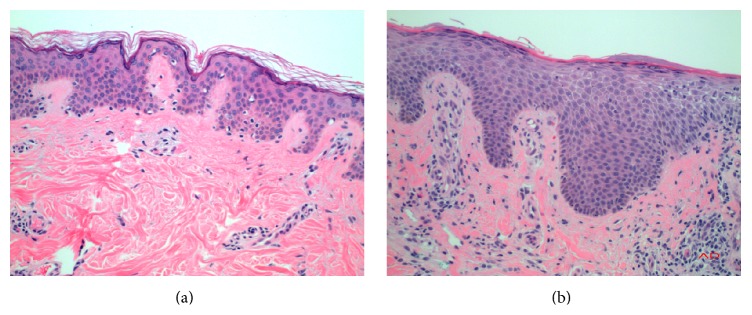
Histology finding of the normal and AD patient's skin. (a) Histology of normal skin. Normal thickness of epidermis (top layer) composed of several layers of squamous cells with the delicate basket-wave keratin (stratum corneum) on the surface. The dermis (bottom part) is composed of sparse fibroblasts with abundant extracellular collagen bundles and embedded capillaries lined by a single layer of endothelial cells (magnification 200x). (b) Histology of subacute spongiotic dermatitis, typically seen in affected skin of AD patients. The epidermis is thickened with slit-like spaces between squamous cells, indicating edema/spongiosis. The overlying basket-wave keratin is replaced by abnormal hyperkeratosis and parakeratosis. The dermis shows increased cellularity composed of mixed inflammatory cells predominantly surrounding small vessels. The inflammatory cells are of predominantly lymphocytes with some mast cells, macrophages, and occasional eosinophils (magnification 200x).

Apart from genetic predisposition, the hallmark pathology of AD is an acute, subacute, or chronic dermatitis of nondistinctive type. The dermal layer contains perivascular or interstitial inflammatory infiltrate composed of many types of inflammatory cells, including plasma cells, mast cells, eosinophils, and B and T lymphocytes. Many types of proinflammatory cytokines are increased in AD patients, such as tumor necrosis factor (TNF) and interleukins (IL-4, IL-9, IL-22), for example [6]. The epidermis often shows edema with spongiosis and increased cell layers with parakeratosis, hyperkeratosis, and dyskeratosis. Stratum corneum, also called basket-wave keratin, the outmost layer of the epidermis normally functioning as the skin barrier, is lost in AD lesions (Figure 1).

**Figure 2:**
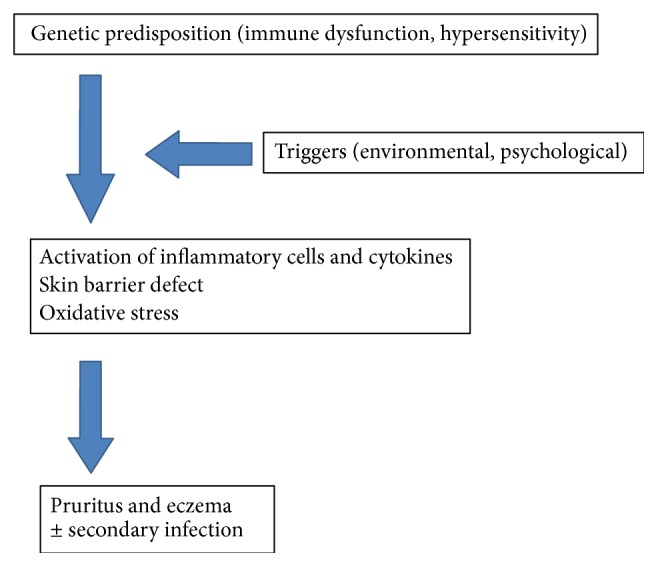
Development and maintenance of atopic dermatitis.

A simplistic version of pathogenesis of AD is illustrated in Figure 2. It is well known that environmental and/or psychological triggers when applied to a genetically predisposed person can initiate skin inflammatory change and destroy intact skin barrier, resulting in clinical manifestations of AD [7, 8]. In recent years, oxidative stress has also been implicated in the pathogenesis of AD.

Oxidative stress is defined as the formation of oxidants in the cells of the human body that acutely or chronically exceeds the antioxidant defense capacity. Oxidants, including free radicals (any species capable of independent existence which contains one or more unpaired electrons) [9], reactive oxygen species (ROS), and nitrogen oxygen species (NOS) and reactive metabolites are produced during normal metabolic activities. Biological antioxidant defense systems exist in cells, including enzyme-based systems (superoxide dismutase, glutathione peroxidase, and peroxiredoxins) and nonenzyme-based systems (vitamins A, C, and E, glutathione, polyphenols, and coenzyme Q10). In excess, the oxidants can react with all cellular macromolecules, including lipids, proteins, nucleic acids, and carbohydrates, particularly polyunsaturated fatty acids on the cell membranes. After the initial reaction with ROS, a chain reaction is started, proceeding to cell injury and, ultimately, cell death [10]. Oxidation metabolites can be quantitatively measured, such as urine or serum nitrate for nitric oxide, malondialdehyde (MDA) for lipid oxidation, and 8-hydroxydeoxyguanosine (8-OHdG) for DNA oxidation [11].

For several decades, there has been increasing evidence linking oxidative stress to several chronic diseases, including cardiovascular disease, diabetes, neurodegenerative disorders, inflammatory diseases, and cancer [12–14]. For example, excess free radicals created through hyperglycemia damage mitochondria, the energy-producing cellular organelle, and are largely responsible for the life-threating complications of type 2 diabetes [14]. It has also been shown that oxidative stress plays an important role in skin aging and development of skin cancer.

It is also well known that oxidative stress promotes tissue inflammation through upregulation of genes that code proinflammatory cytokines. Inflammatory cells in turn release free radicals when activated. Given its prominent inflammatory component, it is conceivable that oxidative stress may play a role in the pathogenesis of AD. The exploration of the association between inflammation and oxidative stress in AD will enhance our understanding of the development and maintenance of the disease, which can be incorporated into formulating new treatment strategies, such as combining anti-inflammatory drugs, immune regulatory agents, skin barrier enhancers, and antioxidants.

In this review, we will summarize available studies exploring the role of oxidative stress in AD and the relationship between oxidative stress and other crucial pathological factors associated with AD. Potential future treatment options will also be discussed.

## 2. Oxidative Stress in Atopic Dermatitis, Involvement, and Possible Mechanisms

**Figure 3:**
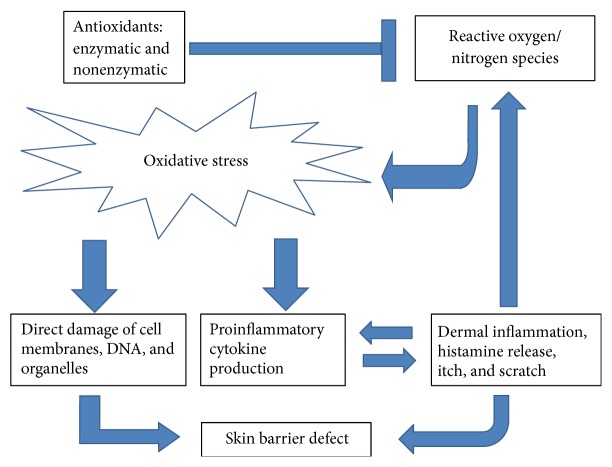
The interplay among oxidative stress, skin barrier defect, and inflammation in atopic dermatitis.

Skin is the largest organ in the human body. It protects the body from external insults, such as chemicals, environment pollutants, and allergens. The skin is therefore a major target of oxidative stress due to reactive species that are constantly generated in the keratinocytes in response to environmental and endogenous prooxidant agents. Physical activity and psychological stress can also create oxidative stress to the skin. Free radicals generated during normal metabolism are an integral part of normal skin function and are usually of little harm because intracellular mechanisms can reduce their damaging effects. However, increased or prolonged free radical action can overwhelm antioxidant defense mechanisms of the skin and contribute to the development of skin disorders, including skin cancer, skin aging, and dermatitis (Figure 3).

Oxidative stress has been implicated in atopic dermatitis for more than 15 years, mainly in the following three aspects: (1) the presence of oxidative stress; (2) increased oxidative stress during AD exacerbation; and (3) decreased antioxidant capability. It was demonstrated that urine markers of oxidative stress are altered in children with AD, including 8-OHdG, nitrite/nitrate and selenium [15]. Those marker levels are higher in children with AD than that in non-AD children. It was suggested that impaired homeostasis of oxygen/nitrogen radicals and increased oxidative stress are involved in the pathophysiology of childhood AD [15]. Chung et al. [16] also found blood antioxidant capacity was significantly less and MDA was higher in preschool children with AD compared to a control group. More recently, Amin et al. and Sivaranjani et al. conducted case-control studies on eczema patients with healthy individuals as controls. They found that, compared to the control group, patients with eczema have a significantly higher level of lipid peroxidation by measuring serum malondialdehyde (MDA), and lower levels of antioxidants including vitamins A, C, and E [17, 18]. Similar findings of the presence of oxidative stress and increased lipid peroxidation were reported in patients with alopecia areata, an inflammatory skin condition closely related to AD [19, 20]. Subsequently, Tsukahara et al. [21, 22] observed oxidative stress and altered antioxidant defenses in children with acute exacerbation of AD. They found that urinary glycosylation end products and bilirubin oxidative metabolites are significantly higher in AD children during hospitalization. Nakai et al. [11] also demonstrated urine nitrate and MDA levels correlate with the severity of AD. Later, Kirino et al. [23] found that heme oxygenase 1, an inducible antioxidant, attenuates the development of atopic dermatitis-like lesions in mice and AD patients. Chung et al. [16] also claimed an association of glutathione-S-transferase polymorphisms with AD risk in preschool age children, implying decreased antioxidant capability may play a role in the pathogenesis of AD. The source of oxidative stress for AD patients could be environmental, physical, and psychological. It is now known that a variety of air pollutants, such as tobacco smoke, volatile organic compounds, formaldehyde, toluene, nitrogen dioxide, and particulate matter act as risk factors and aggravators of AD. Those air pollutants probably induce oxidative stress in the skin, leading to skin barrier dysfunction or immune dysregulation [7]. Song et al. observed increased urine 8-OHdG, a DNA oxidation marker, in children with eczema exposed to short term ultrafine particles [24]. It has been recently shown that the aryl hydrocarbon receptor/aryl hydrocarbon receptor nuclear translocator (AhR/ARNT) signaling system plays an important role in keratinocytes. AhR ligation induces not only oxidative stress but also antioxidant response in a ligand-dependent manner. Environmental pollutants, such as cigarette smoke, bind to AhR and induce ROS production, DNA damage, and inflammatory cytokine production to cause skin inflammation. In contrast, certain flavonoids bind to AhR, resulting in the activation of nuclear factor-erythroid 2-related factor-2 (Nrf2) to produce key molecules that protect cells from oxidative damage [25]. Another source of oxidative stress might be skin microbes. As early as 1970, it was noted that resident flora in AD patients are different from the rest of population. The normal as well as diseased skin of AD patients is markedly colonized with* Staphylococcus aureus*. This may be due to preferential expression of bacterial receptors in AD skin, which may predispose to increased carriage of staphylococci, or defective host defense mechanisms involved in the control of bacterial infection. It was recently shown that increased epidermal fatty acid binding protein is noted and associated with methicillin resistant* Staphylococcus aureus* [26]. The presence of the bacterial pathogen stimulates IL-4 and IgE synthesis to cause dermal inflammation and therefore itching and scratching [27].

Psychological stress, as a social pollutant, is a well-known cause of oxidative stress [8] and causes abnormal skin barrier function in humans [28] and is a frequent cause of AD flares. This may be because psychological stress induces an increase in endogenous corticosteroids, which in turn appears to disrupt not only barrier function but also stratum corneum cohesion as well as epidermal antimicrobial defense [8]. Given the poor sleep pattern, psychosocial burden, and poor quality of life in many AD patients, there has been significant association between AD and depression, namely, a 59% increased likelihood of depression in AD patients. This association could also be linked to neuroinflammatory pathways [29]. Furthermore, recent studies have shown that extraneous physical activity is associated with oxidative stress and increase of proinflammatory mediators [30].

The hallmark of AD is dermal inflammation in affected areas, which could be enhanced by oxidative stress. It is known that oxidative stress can activate nuclear factor kappa-B (NF-*κ*B) pathways to activate gene expression and synthesis of antioxidant enzymes. But the NF-*κ*B pathway activation also induces expression of proinflammatory cytokines, such as IL-6, IL-8, IL-9, and IL-33, which in turn enhances dermal inflammatory infiltrate and histamine release in the affected skin to worsen symptoms [30–33]. In animal experiments, oxidative stress in the skin seems to elicit itching and scratching, even in nonatopic animals. Repeated painting of formaldehyde on the skin of 8-week-old BABL/c mice caused ear swelling and infiltration of inflammatory cells. This was related to the increased expression of IL-4 [34]. But the IL-4 gene expression can be suppressed by antioxidant desferrioxamine treatment [35]. It has also been shown that intradermal hydrogen peroxide can provoke itching through a histamine-independent pathway [36]. These animal studies are suggestive of the possibility that oxidative stress and redox imbalance might develop or aggravate AD by trigging pruritus or enhancing Th2 polarization [7]. On the other hand, inflammation generates high levels of ROS/NOS and other oxidants by activation of several enzymes leading to oxidative stress and cellular damage [30].

Oxidative stress can directly cause damage to epidermal keratinocytes by DNA damage, damage of cellular enzymes, or damage to cell membrane structures through lipid oxidation. These intracellular changes will manifest histomorphologically as epidermal edema/spongiosis and disrupted stratum corneum. One of the most important lipids involved in maintaining an intact skin barrier is the ceramides. These molecules are composed of sphingosine and fatty acid and are produced during keratinization in the stratum corneum (the basket-wave keratin, see Figure 1(a)). The intact epidermal barrier has a key function in limiting the entry of allergens and infectious agents and preventing transdermal water loss. Comparative proteomic profiling has demonstrated that proteins related to skin barrier function (filaggrin-2, corneodesmosin, desmoglein-1, desmocollin-14, and transglutaminase-3) are expressed in significantly lower levels in lesion sites in AD patients [26]. Studies have also shown that the skin barrier is directly damaged by oxidative stress initiated by external pollutants. In a study of 75 adult patients with AD, skin biopsies were taken and dinitrophenylhydrazine (DNP) was measured for the content of carbonyl moieties, a marker of oxidative protein damage. It was noted that DNP formation is significantly increased in AD lesions and correlated with AD severity. It was also observed that DNP is more intense in the superficial layers of the stratum corneum than in the lower layers, indicating the oxidative damage might be attributed to exposure to environmental oxidants. The authors conclude that increased ROS generated from environmental pollutants and solar UV light can induce oxidative protein damage in the stratum corneum, resulting in skin barrier dysfunction and aggravation of AD [37]. It is also observed that exposure of keratinocytes to cigarette smoke will increase the production of hydrogen peroxide, which could induce modification, translocation, and degradation of scavenger receptor B1, a protein that plays an important role in cholesterol trafficking and thereby contributes to the permeability barrier [38]. Furthermore, dermal exposure to m-xylene can induce pathologic change and increase expression of IL-1 alpha and inducible nitric oxide synthase in a rat model [39]. On the other hand, studies have shown that retinoic acid, a vitamin A derivative, and an antioxidant, even at very low levels, can stimulate ceramide production in the epidermis in* in vitro* culture models. Recently, retinoic acid was also shown to be able to downregulate proinflammatory cytokine IL-1 production induced by ultraviolet B radiation [40]. Furthermore, a disrupted skin barrier promotes skin colonization by microbes, and heavy microbial colonization facilitates skin penetration of microbial agents leading to subsequent IgE sensitization [41]. Monocytes from patients with AD are primed to generate ROS in response to zymogens produced by* Staphylococcus aureus *that is heavily colonized on skin of AD patients, leading to damage of the skin barrier by ROS production [42].

## 3. Managing Oxidative Stress in AD

Given the association of oxidative stress with other factors in development and maintenance of AD, it is worthwhile to consider incorporating strategies in reducing oxidative stress in managing AD. This can be accomplished in multiple ways, including reducing free radical production and enhancing antioxidant capacity; diminishing the intensity of inflammation and proinflammatory cytokine production; avoiding environmental, physical, and psychological triggers to achieve prolonged remissions; and applying emollients to maintain the intact skin barrier. The practical approach would be to combine anti-inflammatory agents, immune modulatory drugs, skin emollients, and antioxidants. The antioxidant agents to be considered include melatonin; vitamins A, C, D, and E; oxytocin; and others.

Melatonin is an indolamine mainly produced by the pineal gland [43]. Human skin expresses melatonin receptors (MT1 and MT2). Melatonin has many roles in a variety of physiological functions, such as regulating circadian rhythms for its sleep-inducing activity as well as regulating visual, reproductive, cerebrovascular, and neuroendocrine systems. In addition, melatonin is a powerful endogenous free radical scavenger and functions as a potent anti-inflammatory agent as documented in both* in vitro* and* in vivo* studies [40, 43–45]. It also stimulates some important antioxidant enzymes, such as superoxide dismutase, glutathione peroxidase, and glutathione reductase, to protect cell membranes from lipid peroxidation and neutralizing toxic radicals [43]. Furthermore, melatonin may have important neuroimmunological actions and immunomodulatory effects in allergic diseases. Melatonin has been successfully used in the treatment of cancer, sleep disorders, and aging [43]. In AD patients, melatonin can be used to facilitate a better night's sleep and to reduce skin inflammation. The potential use of melatonin in atopic dermatitis was reported [46]. There are no significant side effects after long-term use; it is safe to use in all ages, including newborns and infants [46].

*Vitamin A*. Vitamin A is a group of chemicals with the same basic bioactive structure. Humans are unable to synthesize vitamin A. The bioactive chemicals of vitamin A can only be obtained from the diet, retinol from animal food sources, and carotenoids from plant sources. They are important in several bodily processes, such as vision, immunity, and hair follicle development, as well as circadian rhythms and in oxidative stress. Vitamin A nuclear receptors are present in the regulatory regions of certain antioxidant enzyme genes in rat livers [47], which are important in the regulation of antioxidant capacity. Vitamin A also has effects on lipid oxidation and may be important in skin health, as lipids are extremely important in maintaining the barrier function of the epidermis [40].

*Vitamin D*. Vitamin D is a steroid hormone that can be produced in the body during a chemical reaction catalyzed by UVB radiation. When there is lack of UV exposure, vitamin D can only be obtained through the diet. The antioxidant capability of vitamin D in skin is not clear. It was shown that, after vitamin D exposure, several antioxidant genes were upregulated in the prostate, including SOD, thioredoxin reductase, and G6PD. Vitamin D was also shown to be able to protect prostate cells from H_2_O_2_-induced cell death [48]. Similar results may be expected in human skin cells. However, no clinical efficacy has been reported so far in AD patients.

*Vitamin E*. Plevnik Kapun et al. [49] found reduced vitamin E concentrations in canine atopic dermatitis. They then divided AD dogs to two groups: one receiving vitamin E supplementation and the other receiving mineral oil as a placebo. The levels of oxidative stress markers showed significant improvement in dogs receiving vitamin E. Similar studies are not currently available for humans, but vitamin E has been used to deter skin aging.

*Oxytocin*. The neuropeptide hormone oxytocin mediates a wide spectrum of tissue-specific actions, ranging from cell growth, cell differentiation, and sodium excretion to stress responses, reproduction, and complex social behaviors. Oxytocin and its receptors are detected in skin keratinocytes and dermal fibroblasts. It appears that it is a novel neuroendocrine mediator in human homeostasis and clinically relevant to stressed skin conditions such as AD. It is postulated that, in AD patients, the oxytocin system is deregulated in terms of cellular proliferation, inflammation, and response to oxidative stress. Oxytocin receptor reduction in dermal fibroblasts and keratinocytes leads to elevated levels of reactive oxygen species and reduced levels of glutathione. Those keratinocytes also exhibited an increased release of the proinflammatory cytokines, such as IL-6 [50].

Other methods used in treatment of AD also apply their antioxidant capabilities, one of which is coal tar. Coal tar has been around more than 200 years. It consists of more than 10,000 chemical compounds. Until recently, the molecular mode of action was obscure. A recent study demonstrated that coal tar induces AhR-dependent skin barrier repair by inducing epidermal gene and protein expression including filaggrin in AD patients [51]. Topical application of coal tar is an effective skin AD therapy for reducing inflammation and itch. Another example is hydrogen water. Yoon et al. fed mice with AD with hydrogen water, a potent and harmless antioxidant, and showed positive effect in relieving AD [52]. Wiegand et al. tested a zinc oxide- (ZnO-) functionalized textile for its skin-protective effects in AD patients [53]. In addition to possessing very good biocompatibility and being well tolerated by AD patients, rapid improvement of AD severity, pruritus, and subjective sleep quality were observed in AD patients wearing this type of textile. The authors attributed the success to the high antioxidative capacity of the ZnO textile and its strong antibacterial activity.

Although theoretically promising, dietary antioxidant supplement has not shown significant clinical benefit [54]. But most studies have been small in scale with low numbers of participants and poor quality control. Although most dietary supplements have no side effects to health, high dose vitamin D has been implicated in causing serious medical problems. The cost of long-term use of supplements is also a concern. Additional large scale and well-designed studies are needed to fully evaluate the efficacy in AD. A holistic approach would encompass assessment of the severity and impact on quality of life, assessment and management of environmental physical and psychological triggers, recognition and treatment of infection, and restoration of the skin barrier function [55].

## 4. Conclusion

Oxidative stress appears to be one of the important factors in the pathogenesis of atopic dermatitis. It not only directly damages the cellular structures of the skin but also enhances dermal inflammation and weakens the skin barrier function and enables infections by microbial pathogens. Given our current understanding of the pathogenesis of AD, strategies should be focused on multimodality and individualized therapy. Treatment goals should include (1) reducing environmental insults and psychological stress; (2) enhancing the skin barrier function by skin hydration and emollients; (3) exploring anti-inflammatory and immune modulatory agents as second-line therapy; and (4) using oral antioxidant supplements, such as appropriate amount of daily vitamins and melatonin. At this time, well-designed clinical studies are needed to fully evaluate those approaches for the ultimate goal of not only relieving symptoms but also improving the overall quality of life in AD patients.

## Abbreviations

AD: Atopic dermatitis
Ig: Immunoglobulin
MDA: Malondialdehyde
ROS: Reactive oxygen species
NOS: Nitrogen oxygen species
8-OHdG: 8-Hydroxydeoxyguanosine.
